# Understanding the Health Literacy in Patients With Thrombotic Thrombocytopenic Purpura

**DOI:** 10.1097/HS9.0000000000000462

**Published:** 2020-08-11

**Authors:** Leydi C. Velasquez Pereira, Bogac Ercig, Kadri Kangro, Matthieu Jamme, Sandrine Malot, Lionel Galicier, Pascale Poullin, François Provôt, Claire Presne, Tarik Kanouni, Aude Servais, Ygal Benhamou, Nicolas Daguindau, Karen Vanhoorelbeke, Elie Azoulay, Agnès Veyradier, Paul Coppo

**Affiliations:** 1 Laboratory for Thrombosis Research, IRF Life Sciences, KU Leuven Campus Kulak Kortrijk, Kortrijk, Belgium; 2 Department of Plasma Proteins, Sanquin-Academic Medical Center Landsteiner Laboratory, Amsterdam, the Netherlands; 3 Médecine Intensive Réanimation-Néphrologie, CH de Poissy Saint Germain en Laye, Poissy, Centre de Recherche en Epidémiologie et Santé des Populations, INSERM U1018, Paris-Sud University, Villejuif, France; 4 Centre de Référence des Microangiopathies Thrombotiques, AP-HP.6, Paris, France; 5 Department of Clinical Immunology, Hôpital Saint-Louis, AP-HP, Université Paris-Diderot, Paris, France; 6 National Reference Center for Castleman Disease (CRMdC), Paris, France; 7 EA3518, Université Paris-Diderot, Paris, France; 8 Department of Apheresis, Regional Reference Center for Thrombotic Microangiopathy, Aix-Marseille University, CHU de Marseille-Hôpital de la Conception, Marseille, France; 9 Service de Néphrologie, Hôpital Albert Calmette, Lille, France; 10 Service de Néphrologie, Hôpital Sud, CHU Amiens, Amiens, France; 11 Service d’Hématologie, CHU Saint Eloi, Montpellier, France; 12 Service de Néphrologie–Dialyse Adulte, Hôpital Necker-Enfants Malades, AP-HP, Paris, France; 13 Service de Médecine Interne, U1096, UNIROUEN, Normandie Universitaire, Rouen, France; 14 Service d’Hématologie, CH d’Annecy, France; 15 Medical ICU, Saint-Louis Hospital, AP-HP, ECSTRA Team, and Clinical Epidemiology, UMR 1153 (Center of Epidemiology and Biostatistics, Sorbonne Paris Cité, CRESS), INSERM, Paris Diderot Sorbonne University, Paris, France; 16 service d’Hématologie Biologique, Groupe Hospitalier Saint-Louis-Lariboisière, AP-HP, Université Paris-Diderot, Paris, France; 17 EA3518 Recherche Clinique en Hématologie, Immunologie et Transplantation, Équipe Microangiopathies Thrombotiques, ADAMTS13 et Facteur Willebrand, Institut de Recherche Saint-Louis, Université Paris-Diderot, Paris, France; 18 service d’Hématologie et Sorbonne Université, AP-HP.6, Paris, France; 19 Centre de Recherche des Cordeliers, INSERM, Sorbonne Université, USPC, Université Paris Descartes, Université Paris-Diderot, Paris, France.

## Abstract

Following an acute thrombotic thrombocytopenic purpura (TTP) episode, patients are at risk for relapse, and a careful long-term follow-up is needed. Adherence to the follow-up by patients implies a good understanding of the disease. However, TTP literacy in patients is currently unknown. To explore the TTP literacy in patients and identify factors associated with poor disease understanding, a questionnaire was developed focusing on patient's characteristics, knowledge about TTP and patients’ actions in an emergency. The questionnaire was presented to 120 TTP patients in remission from the French National Registry for Thrombotic Microangiopathies. TTP literacy was low in 24%, intermediate in 43% and high in 33% of the patients. Low TTP literacy was associated with older age and low education level. Among the knowledge gaps identified, few patients knew that plasma exchange in acute phase is mandatory and has to be done daily (39%), 47% of participants did not consider themselves at risk for relapse, and 30% of women did not know that pregnancy exposes them to a greater risk of relapse. Importantly, few patients responded about life-saving actions in an emergency. Hence, the design of educational material should pay special attention to the age and education level of the target population focusing on the events leading to TTP, the importance of the emergency treatment, controllable predisposing factors for TTP development and patient attitude in an emergency.

## Introduction

Thrombotic thrombocytopenic purpura (TTP) is a life-threatening disease characterized by microangiopathic hemolytic anemia, a profound thrombocytopenia and organ failure of variable severity. TTP results from a severe deficiency in the Von Willebrand factor (VWF)-cleaving protease ADAMTS13 (A Disintegrin And Metalloprotease with Thrombospondin type 1 motif, member 13), either due to mutations in the *ADAMTS13* gene (congenital TTP, cTTP), or due to circulating anti-ADAMTS13 autoantibodies (immune-mediated TTP, iTTP).^1^ The annual incidence rate of cTTP is estimated at 1 case per million people, while iTTP incidence is estimated 4 to 10 cases per million people.^2–4^ In the absence of treatment, TTP is almost always fatal (mortality rate >90%); however, when treated on time with plasma infusion (cTTP) or plasma exchange and immunosuppressive agents (iTTP), the mortality decreases to 10% to 15%.^5,6^ Even with optimal management, neurological and physical sequels may persist.^7,8^ A persistent, severe ADAMTS13 deficiency following the acute phase exposes patients to a high risk of relapse within a variable period of time ranging from weeks to years,^9–11^ and patients may develop additional autoimmune diseases.^12^ Consequently, a long term follow-up is recommended for all patients.^13^

The rarity of TTP leads to misdiagnosis,^1,6,14^ which may obscure the prognosis of the disease and if left untreated can lead to death, especially in the most severe cases with cardiac or cerebral involvement.^15^ Therefore, campaigns to raise awareness in the medical community are mandatory. Regarding creating TTP awareness for patients, several TTP patient support groups (www.ttpcommunity.be, www.answeringttp.org, www.ttpnetwork.org.uk and www.ouhsc.edu/platelets) exist and offer pedagogic material about TTP, testimonies from other patients who are affected with the disease, and specialist assistance.^16,17^ Moreover, the portal Orphanet (www.orpha.net/consor) provides information about rare diseases, both for physicians and for patients. In total, these sources of information contribute to health literacy in TTP, a term that refers to the “degree to which individuals are able to obtain, process and understand health information in order to orientate health decision.”^18^ A better understanding of the disease and treatment can help patients to increase their involvement in the global management of the disease and to improve the quality of assistance, with better compliance to recommendations and earlier detection of symptoms to maximize physician resources.^19^ In this way, healthcare providers need to be aware of patients’ knowledge deficits to develop more effective education strategies. Such studies have been performed for patients who have chronic diseases, or are in intensive care units.^20–25^ However, it is still unknown how much TTP patients understand their disease. For this reason, the current study investigated TTP literacy using a questionnaire. The outcome of this questionnaire allowed us to assess how much the patients understand the disease and to identify factors associated with poor comprehension of TTP.

## Materials and methods

### Design and population

In this observational, analytical study, participants were recruited for 3 years (ie, between 2016 and 2019) from the 13 main centers of the French Reference Center for thrombotic microangiopathies (CNR-MAT, see Contributors section), using a non-probability convenience sample. On each center, all patients available during this period were invited non-selectively to take part in the study either at the time of their scheduled follow-up appointment or by phone. This study was part of the Thrombotic Microangiopathy program study approved by our institutional review board (CPP04807) in accordance with the Declaration of Helsinki, and the French Data Protection Authority (“Commission Nationale Informatique et Libertés,” CNIL, authorization no. 911539, and “Comité consultatif sur le traitement de l’information en matière de recherche dans le domaine de la santé,” CCTIRS, authorization no. 11.537, Paris, France).

### Questionnaire

The questionnaire consisted of 35 questions (Supplemental Table 2). In the first question (Q1), the participant's name was requested. Forms were anonymized for analysis. Names were only used at the end of the study by the study nurse (SM) to further consult the medical record of the patient regarding how many episodes of TTP the patient had, and if there was a central nervous system involvement (neurologic symptoms, confusion, convulsion, coma, Glasgow score, and focal neurologic deficit) in the last acute episode of the disease. The next questions were divided into three groups. The first group consisted of 10 questions and were (i) related to the characteristics of the participants such as age (Q2), gender (Q3), geographic origin (Q4), profession (Q5), languages spoken fluently (Q6), education level (Q7), or (ii) to the disease such as when the patient started feeling sick (Q8), date of TTP diagnosis (Q9), being a member of a patient association (Q10), and use of Orphanet (Q11). The second group of questions consisted of 21 questions that were designed to get insight into the patient knowledge about the disease (TTP literacy). The 21 questions were either related to “what type of disease is TTP?” (Q12–Q14), to “which abnormalities in the body cause TTP?” (Q15–Q21), “what is the treatment for the disease?” (Q22–Q25), and to “what is the risk of relapse and what triggers TTP?” (Q26–Q32). The third group contained three questions where the patients were asked what they would do when they thought they had a relapse or when they felt numbness (Q33), if anything was unclear related to their disease (Q34), and if they had any additional questions (Q35).

The questionnaire was designed by LCVP, BE, KK under the supervision of PC and KV, and was next sent to all clinicians involved in this study for review. Based on the feedback, the questionnaire was slightly adapted. All clinicians involved in the study approved the final version of the questionnaire.

### Scoring of the questions

TTP literacy was analyzed by scoring only the second group of questions (Q12–Q32), using open-ended questions (Q15 and Q22), and closed-ended questions with two-options (Q12-13, Q19–Q20, Q26-27, Q29) and multiple-choice answers (Q14, Q16–Q18, Q21, Q23–Q25, Q28, Q30–Q32; Supplemental Table 2). The correct answer for the open-ended question inquiring which protein is missing in TTP (Q15) as well as the correct response to the questions with two-option answers (Q12-13, Q19–Q20, Q26-27, Q29), obtained 1 point; an incorrect answer, no answer, or not knowing the answer received 0 points (Supplemental Table 1). The question Q22, “what is the main treatment for TTP?” was scored as 1 when “plasma exchange with or without another treatment” was the answer while 0.5 points were given when only “rituximab” or “corticosteroids” (without mentioning plasma exchange) were given as an answer. The reason behind this scoring was that although rituximab and corticosteroids are not the main treatment for TTP; they are used to treat TTP. The questions with multiple-choice answers and only one correct option (Q16–Q18, Q23, Q28, and Q30–Q31) provided 1 point. While for Q21, Q24 and Q25, where the correct answer consisted of two answers, the correct answer received 1 point, and 0.5 points were given for the individual correct answers. In addition, for Q32 where the correct answer was composed of three possible answers (received 1 point), 0.3 points were given for each individual correct answer. The distribution of points was different for Q14, “Thrombotic thrombocytopenic purpura is,” 1 point was given to the selection of either “an autoimmune condition,” “a genetic condition,” or selection of both responses. The rationale behind this was that the participants of the study had iTTP; thus, it was expected that the vast majority of them would reply that TTP is an autoimmune condition.

### Scoring TTP literacy

To score TTP literacy on “what type of disease is TTP?” (Q12–Q14), all points of Q12–Q14 were added up, multiplied by 5, and divided by 3 (the number of “what type of disease is TTP?” questions). The same calculation was applied for the questions related to ‘which abnormalities in the body cause TTP?’ (Q15–Q21), “what is the treatment for the disease?” (Q22–Q25), and “what is the risk of relapse and what triggers TTP” (Q26–Q32) to score the TTP literacy related to each question group. This scoring system led to scores ranging from 0 to 5. The scores were arbitrarily defined as low (scores <3); intermediate (scores ≥3…<4) and high (scores ≥4…5). Finally, to score the overall TTP literacy, all points from the second group of questions were added up, multiplied by 5 and divided by 21.

### Identifying the specific aspects of TTP that are well or poorly understood

To identify the specific aspects of TTP that are well or poorly understood by the patients, the percentage of patients giving a correct (1 point; Supplemental Table 1), partially correct (>0 but <1 points; Supplemental Table 1), wrong (0 points; Supplemental Table 1), not knowing the answer (0 points; Supplemental Table 1) or no answer (0 points; Supplemental Table 1) to Q12–Q32, were calculated. The knowledge about each question was classified as low when less than 60% of the participants correctly answered the question, intermediate between 60% and 80% of patients with a correct answer, and high when more than 80% of the participants had a correct answer.

### Statistical analysis

EPIDAT 4® was used to calculate the sample size for the present study considering the prevalence of TTP in France, 13 cases per million people,^37^ with a confidence level of 95% and 8% margin of error. IBM SPSS statistics for Windows, version 19.0 (IBM Corp., Armonk, NY, USA) was used for statistical analyses. Time since TTP diagnosis was calculated from the date of TTP diagnosis (Q9) to the date that the questionnaire was filled. Mean values with range or standard deviation, or median with the 10 to 90th percentile are provided for quantitative values; for qualitative values, frequencies and percentages were calculated. To measure the differences in quantitative variables, Student's *t* test was used while the categorical variables were analyzed using one-way ANOVA. The association of age with TTP literacy was measured using a Pearson correlation and a Spearman correlation was used to analyze the influence of time since TTP diagnosis and TTP literacy. Multivariate linear regression was applied for the analysis of the independent association of the variables that were found to be significant in the univariate analysis. A *p value* of <0.05 was considered statistically significant for all tests.

## Results

### Characteristics of the study cohort

One hundred thirty-eight TTP patients were approached during the 3-year recruitment period; 120 of these agreed to participate, and 18 (13%) did not participate in the study. All 18 patients were contacted by phone by our study nurse (Sandrine Malot, SM) a first time, and then a second time some weeks later as no response was obtained. Hence, patients who did not respond after two solicitations were not further considered for the study. Analysis of the clinical record from the non-participants showed that all 18 patients have recovered from iTTP for years, and all have a spaced follow-up. Additionally, none of the non-participants had a relevant background characteristic that could influence the participation in the study, i.e. refugee, homeless or had a language barrier. Patients agreeing to take part prospectively in the study were interviewed either by their clinician or by the study nurse (SM) of the CNR-MAT, during a follow-up appointment or by phone. Characteristics of the study cohort are summarized in Table 1. The mean age of the study population was 44 years (18–85 years). The majority of participants were females (75%). Most of the participants were from Europe (76%) and spoke French fluently (97%). The highest education level of the majority of the study participants was high school (47%). Moreover, the median time since the participants had been diagnosed with TTP was 5 years (1–14 years). A low percentage of the population was a member of a patient association (2%) or used the website Orphanet (7%). The clinical record of the patients showed that most of the participants suffered only one episode of the disease (73%). In addition, information regarding central nervous system involvement in the last acute episode of the disease was available for 78 of the participants; among them, half (54%) experienced focal neurologic signs, including confusion (27%), convulsion (1%), coma (1%), focal neurological deficit (33%) or had a Glasgow score lower than 14 (1%).

**Table 1:**
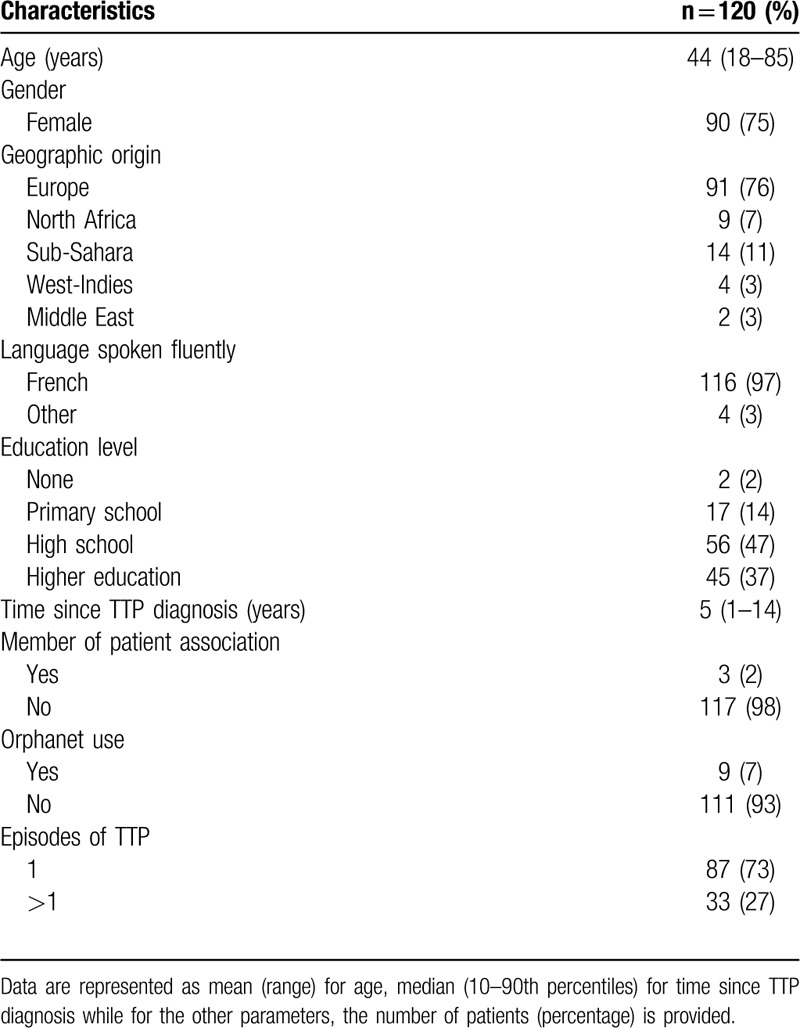
Characteristics of the Study Participants.

The question “profession” was included in the questionnaire (not shown) to know whether any patient had a biomedical background as this might influence the outcome of the TTP literacy. However, only three patients had a biomedical background. Question about when the patient started feeling sick was included (not shown) to evaluate the influence of the timely diagnosis on TTP literacy. However, patients were not sure about the date of their first symptoms of the disease, and it was not possible to confirm that information in the clinical records. Therefore, this question was excluded from the analysis.

### TTP literacy score

TTP literacy was evaluated by scoring the second group of questions that investigated the knowledge on “what type of disease is TTP?” (Q12–Q14), “which abnormalities in the body cause TTP?” (Q15–Q21), “what is the treatment for the disease?” (Q22–Q25), and “what is the risk of relapse and what triggers TTP?” (Q26–Q32). The resulting scores show that the majority of patients (73%) had high knowledge about “what type of disease is TTP?” (score ≥4…5), low knowledge (score <3) about “which abnormalities in the body cause TTP?” (40% of the patients), and intermediate knowledge (score ≥3…<4) regarding “what is the treatment for the disease?” (46% of the patients), and “what is the risk of relapse and what triggers TTP?” (40% of patients). The overall TTP literacy score was intermediate in 43% of the patients, high in 33% of the patients and low in 24% of the patients (Table 2). No statistically significant differences were observed between the center of recruitment of the patients and TTP literacy score as determined by one-way ANOVA (f(8,104) = 1.55, p value = 0.15). Moreover, the comparison of the literacy score between centers according to the number of patients revealed no statistical difference either (r = 0.2641, p = 0.3832) (ie, the literacy score of the centers was not correlated to their volume of patients).

**Table 2:**
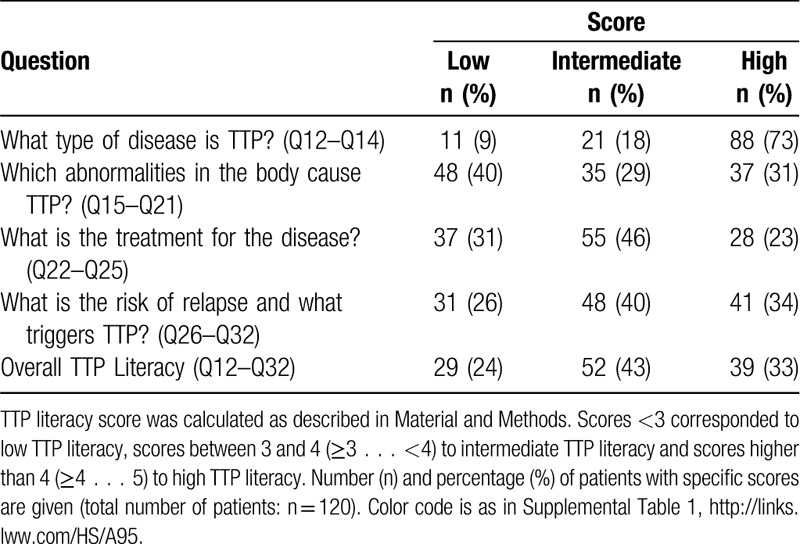
TTP Literacy Score.

### Understanding of specific aspects of TTP

**Figure 1:**
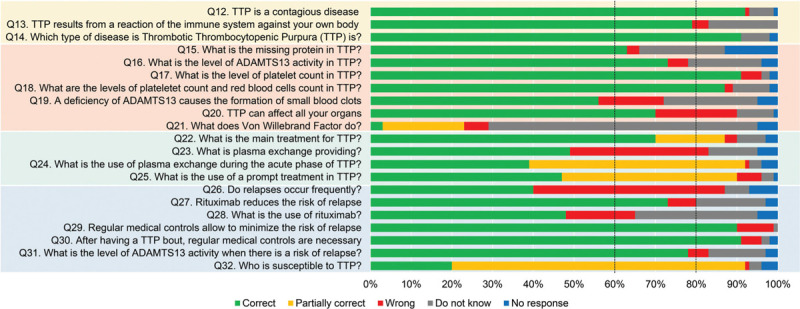
**Overview of specific aspects of TTP that are well or poorly understood.** The percentages of patients giving a correct answer to the questions are depicted in green, a partially correct answer in yellow, a wrong answer in red, “Do not know” answer in grey, and no response in blue. It was defined that the knowledge of the specific aspect of TTP was low when less than 60% of the patients correctly answered the question (dotted line), was intermediate when between 60% and 80% of patients gave a correct answer (dotted line) and was high if more than 80% of the patients gave a correct answer. A complete presentation of the questions and answers is detailed in the Supplemental Table 1 as well as the description of the questions color code.

Next, we analyzed per individual question the percentage of patients that gave a correct (1 point), partially correct (>0 but <1 points), wrong (0 points), not knowing the answer (0 points) or no answer (0 points) (Fig. 1, Supplemental Table 1), to identify which specific aspects of TTP are well or poorly understood by the patients on “what type of disease is TTP?” (Q12–Q14), “which abnormalities in the body cause TTP?” (Q15–Q21), “what is the treatment for the disease?” (Q22–Q25), and “what is the risk of relapse and what triggers TTP?” (Q26–Q32). This analysis shows that the understanding of questions related to “what type of disease is TTP?” (Q12–Q14; Fig. 1) was relatively high as more than 79% of the patients answered the questions correctly (Fig. 1). Analyzing the answers to the questions assessing the level of knowledge on “which abnormalities in the body cause TTP?” (Q15–Q21; Fig. 1) shows that the participants understood well that platelet and red blood cell counts are decreased in TTP (Q17, Q18; Fig. 1). On the other hand, comprehension of the role of ADAMTS13 in TTP was mostly intermediate since between 56% and 80% of the patients could answer these questions correctly (Q15, Q16, Q19, Q20; Fig. 1). The role of VWF in TTP was the least well known (Q21), as only 23% of the patients gave a correct or partially correct answer (Fig. 1). The analysis of the knowledge on “what is the treatment for the disease?” (Q22–Q25; Fig. 1) shows that 70% of the participants knew that plasma exchange is the main treatment for TTP (Q22). However, why plasma exchange is efficient as a treatment and when it is needed, was less clear since less than 60% of the patients gave a correct answer (Fig. 1). Analyzing the comprehension of “what is the risk of relapse and what triggers TTP?” (Q26–Q32; Fig. 1) clearly shows that more than 78% of the patients knew that a regular medical follow up is needed to prevent relapse and that this is linked with ADAMTS13 activity (Q29–Q31). Moreover, 73% of the patients knew rituximab reduces the risk of relapse (Q27), and 48% of the patients knew the function of rituximab as a modulator of the immune response (Q28). Patients perception of how frequent relapses occur in TTP is relative to previous patient experience with the disease. However, only 40% of the patients knew that relapses frequently occur in the absence of regular follow-up and preemptive strategy (Q26). Finally, only 20% of the patients were aware that TTP is triggered by pregnancy, that it is more common in females, and that it is a rare disease (Q32). However, 44% of the female participants answered that TTP is more common in women, and 71% of the women younger than 40-year-old considered pregnancy as a precipitating factor for TTP in the answer, providing evidence that correct answers are more frequently provided when the issue directly impacts patients.

### Age and education level are associated with TTP literacy

Analysis of possible factors associated with poor comprehension of the disease by TTP patients was done by exploring characteristics of the participants that are related to age, gender, geographic origin, spoken languages, education level, time since TTP diagnosis and the number of TTP episodes (Table 3). Although the socio-economic status (income) can also influence health literacy, this variable could not be provided here. Nevertheless, inspection from the clinical record from the participants showed that all of them had social insurance, and none of the participants was refugee nor homeless. Here, education level was therefore used as a reliable and feasible feature to address the socio-economic status. After performing a univariate analysis, a statistically significant association was found with age, geographic origin, education level and the number of TTP episodes (p value <0.05). We found a negative linear relationship between age and TTP literacy: for one year aged, TTP score would be reduced by 0.3 points (p value <0.05). Participants from the Sub-Sahara and Middle East regions had the lowest literacy score. Additionally, high education level and >1 episode of TTP contributed positively to the literacy score. Finally, the influence of central nervous system involvement (neurologic symptoms, confusion, convulsion, coma, Glasgow score, and focal neurologic deficit) in the last episode of TTP and TTP literacy was analyzed using the information available for 78 patients. However, no statistically significant differences were detected after multivariate analysis (f (5, 72) = 1.37, p value = 0.24, R^2^ = −0.09).

**Table 3:**
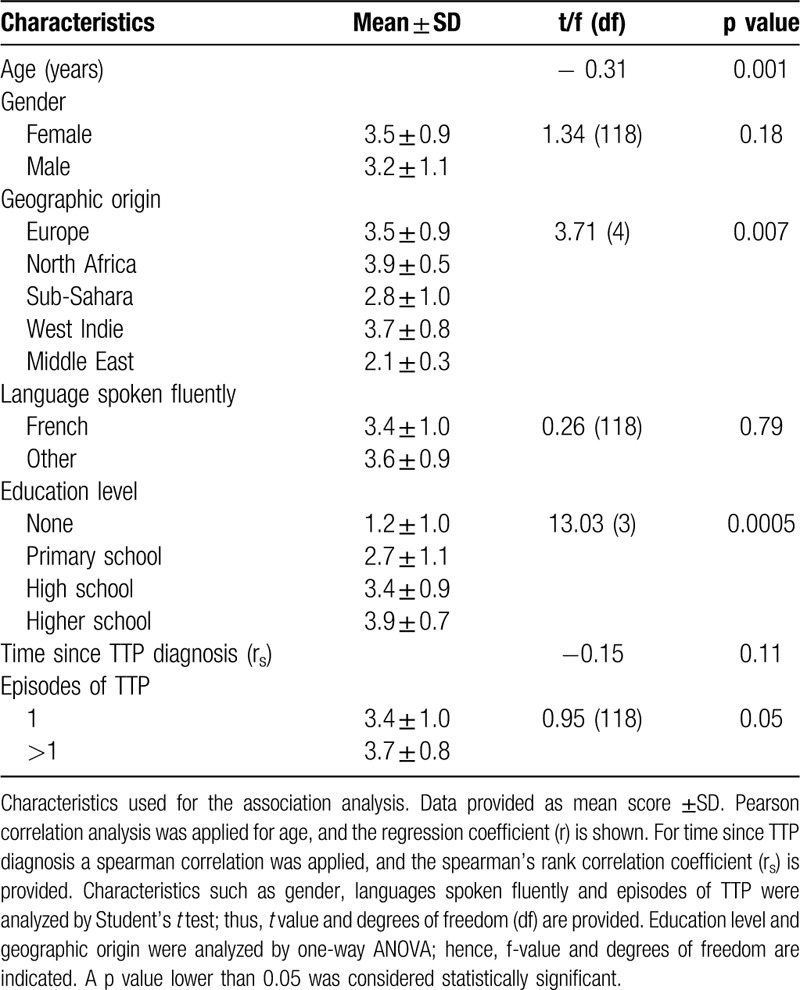
Factors Predicting Low Literacy Score by TTP Patients (Univariate Analysis, n = 120)

Next, to confirm that age, geographic origin, education level and the number of TTP episodes were independently associated with TTP literacy, multivariate linear regression models were performed (Table 4). The analysis of the variables together in the regression model showed that the geographic origin and the number of TTP episodes were no longer statistically significant. Taken together, these results indicate that age and education level are the main factors that affect TTP literacy.

**Table 4:**
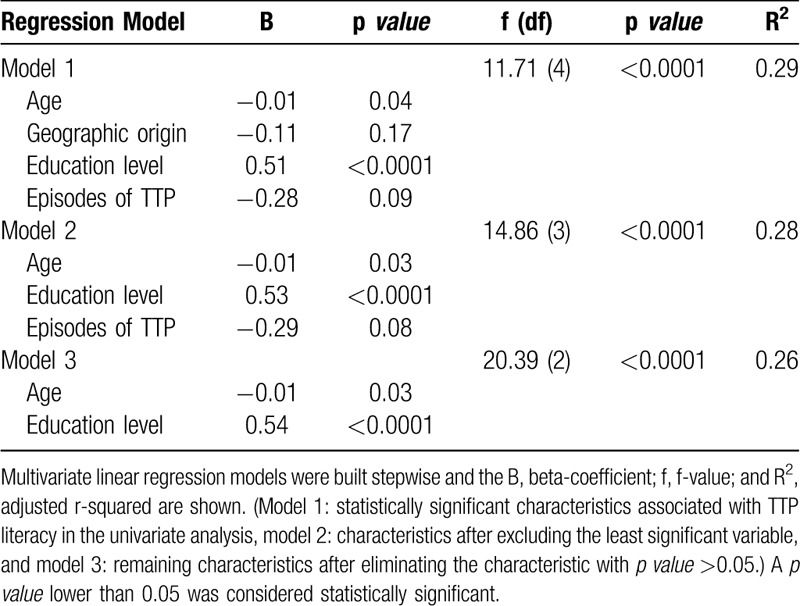
Factors Predicting Low Literacy Score by TTP Patients (Multivariate Analysis, n = 120)

### Questions related to actions to be taken in an emergency setting and patient concerns

In the last group of questions, only a quarter of participants (33/120) replied. Among the responders actions in an emergency setting are directed towards immediate contact with the physician or attendance at the hospital, and undergo a blood test. Regarding the additional comments from the patients, 9% of the patients consider TTP a complex disease and among the expressed concerns were “what causes TTP?” (37%), “why the disease has no definitive cure?,” “is it possible to cure the disease definitely?” (15%), “can I get other diseases because of having TTP?” (12%), “is there anything I can do to avoid getting sick again?” (9%), “why me?” (6%), and one participant asked “why did I receive treatment only when I am sick and not when I am healthy?” Additionally, one patient showed an intention to be involved in patient association groups, and another was interested to know the results from the current research. Only one participant expressed “the less I know the better.”

## Discussion

In this pioneering study exploring the health literacy in TTP patients, we report that our patient cohort had substantial knowledge about TTP. Likewise, we identified older age and basic education level as critical factors associated with less comprehension about TTP. In line with this statement, age and education level have been reported to influence disease comprehension in patients from intensive care units and patients with chronic conditions such as asthma, diabetes, congestive heart failure, hypertension and cancer.^20–26^ These observations may result from a limitation for communication due to the education level.^21^ In addition, the elderly are less likely to be actively involved in getting more information about the disease since they rely on the expertise of the health professionals.^26^ Moreover, elderly may have more limited access to the most modern information systems.^26,27^

For this study, we used a large sample size considering the low prevalence of TTP.^1^ Importantly, most patients from the participating centers agreed to participate in the study, rending our patients highly representative of our TTP population by limiting recruitment bias. However, the TTP literacy in our cohort of patients could be overestimated compared to the general TTP population as our patients were recruited from health care providers belonging to the French Reference Center for thrombotic microangiopathies that usually recruits at least 10 TTP patients per year. This implies that these hospitals are trained to manage TTP, and TTP patients are informed about their condition. Moreover, all participants have been invariably enrolled in various observational studies. Hence, they all have received a minimal (although not standardized) set of information regarding the need to understand better the epidemiology of the disease at the national level, the factors associated with prognosis and response to treatment, as well as long term outcome. Nevertheless, it was unclear the degree at which patients understand the information provided. Additionally, considering that participants belong to different reference centers, it is likely that the information about TTP varies substantially from one practitioner (and from one center) to the other. We report that TTP literacy was intermediate or high in a majority of patients and comparable among the recruitment centers.

Detailed analysis of the responses showed that patients were fairly aware of “what type of disease is iTTP?.” The knowledge about “which abnormalities in the body cause TTP?” was low and it was not clear for all patients that ADAMTS13 is the protein with decreased activity in TTP that leads to the formation of blood clots in different organs of the body,^1^ despite its essential impact in treatment and long-term follow-up. As it could have been expected, participants failed to identify VWF as the other crucial factor for the disease, probably because no therapeutic action or assessment is specifically directed against this protein. However, with the recently available anti-VWF agent caplacizumab (that interferes with the interaction between VWF and platelets) in the therapeutic arsenal,^28^ this protein could now be considered as a crucial therapeutic target by patients, in the same way as ADAMTS13 and anti-ADAMTS13 antibodies. In this aspect, it is all the more true that patients may require many weeks of this treatment following clinical remission achievement, in hospital but also in outpatient care (ie, at home). Regarding “what is the treatment for the disease?,” patients usually considered correctly plasma exchange as the cornerstone treatment of TTP at the acute phase, although the importance of prompt treatment and the frequency of plasma exchange were less clear.^29^ In addition, we observed that patients’ answers reflected unavoidably their personal experience. For example, rituximab, which was still used as a salvage therapy at the time of patients’ recruitment, was typically considered as a standard frontline therapy for patients who experienced a refractory disease or an exacerbation.^29–31^ These discrepant answers reflect a need from practitioners to clarify better the role of rituximab in the therapeutic arsenal of iTTP. Regarding follow-up and the assessment of the relapse risk, patients were aware that TTP is a serious condition; in this way, they related the importance of visiting a medical center as soon as they observed the presence of petechia, fatigue or symptoms of circulatory occlusion. Furthermore, participants usually underestimated the risk of relapse, even though they were aware that regular medical controls allow minimizing this risk. Lastly, patients were globally unaware of the controllable key factors associated with relapse, especially pregnancy.^32–34^ Finally, an important finding of this work is that a high proportion of patients did not answer the question regarding what to do in case of an emergency setting. This finding challenges our information and needs efforts from practitioners to clarify the requested actions in case of unusual symptoms including bleeding and neurological symptoms, but also unusual fatigue. In clinical practice, patients should be educated to perform a blood cell count rapidly in such context and contact their general practitioner in an emergency if abnormal values are present or if symptoms persist.

In conclusion, investing in explaining the pathophysiology of TTP, the importance of prompt treatment, the latent risk of relapse, controllable predisposing factors for TTP development and actions to be taken by the patient in case of an emergency scenario; should improve TTP literacy in patients. Future studies considering educational interventions and local human resources assigned to TTP management (consultation specially assigned to TTP, educational nurse, duration of follow-up visit), will then reveal how patient education helps to improve adherence to follow-up and practitioners’ recommendations which is expected to decrease the associated morbidity and additional costs to the health system,^7,35^ and improve patients’ quality of life.^36^

## Supplementary Material

Supplemental Digital Content

## Appendix

**The members of the Reference Center for Thrombotic Microangiopathies (CNR-MAT) are:** Augusto Jean-François (Service de Néphrologie, dialyse et transplantation; CHU Larrey, Angers); Azoulay Elie (Service de Réanimation Médicale, Hôpital Saint-Louis, Paris); Barbay Virginie (Laboratoire d’Hématologie, CHU Charles Nicolle, Rouen); Benhamou Ygal (Service de Médecine Interne, CHU Charles Nicolle, Rouen); Bordessoule Dominique (Service d’Hématologie, Hôpital Dupuytren, Limoges); Charasse Christophe (Service de Néphrologie, Centre Hospitalier de Saint-Brieuc); Charvet-Rumpler Anne (Service d’Hématologie, CHU de Dijon); Chauveau Dominique (Service de Néphrologie et Immunologie Clinique, CHU Rangueil, Toulouse); Choukroun Gabriel (Service de Néphrologie, Hôpital Sud, Amiens); Coindre Jean-Philippe (Service de Néphrologie, CH Le Mans); Coppo Paul (Service d’Hématologie, Hôpital Saint-Antoine, Paris); Corre Elise (Service d’Hématologie, Hôpital Saint-Antoine, Paris); Delmas Yahsou (Service de Néphrologie, CHU de Bordeaux, Bordeaux); Deschenes Georges (Service de Néphrologie Pédiatrique, Hôpital Robert Debré, Paris); Devidas Alain (Service d’Hématologie, Hôpital Sud-Francilien, Corbeil-Essonnes); Dossier Antoine (Service de Néphrologie, Hôpital Bichat, Paris); Fain Olivier (Service de Médecine Interne, Hôpital Saint-Antoine, Paris); Fakhouri Fadi (Service de Néphrologie, CHU Hôtel-Dieu, Nantes); Frémeaux-Bacchi Véronique (Laboratoire d’Immunologie, Hôpital Européen Georges Pompidou, Paris); Galicier Lionel (Service d’Immunopathologie, Hôpital Saint-Louis, Paris); Grangé Steven (Service de Réanimation Médicale, CHU Charles Nicolle, Rouen); Guidet Bertrand (Service de Réanimation Médicale, Hôpital Saint-Antoine, Paris); Halimi Jean-Michel (Service de Néphrologie Pédiatrique, Hôpital Bretonneau, Tours); Hamidou Mohamed (Service de Médecine Interne, Hôtel-Dieu, Nantes); Herbrecht Raoul (service d’Oncologie et d’Hématologie, Hôpital de Hautepierre, Strasbourg); Hié Miguel (Service de Médecine Interne, Groupe Hospitalier Pitié-Salpétrière, Paris); Jacobs Frédéric (Service de Réanimation Médicale, Hôpital Antoine Béclère, Clamart); Joly Bérangère (Service d’Hématologie Biologique, Hôpital Lariboisière, Paris); Kanouni Tarik (Unité d’Hémaphrèse, Service d’Hématologie, CHU de Montpellier); Kaplanski Gilles (Service de Médecine Interne, Hôpital la Conception, Marseille); Lautrette Alexandre (Hôpital Gabriel Montpied, Service de Réanimation médicale, Clermont-Ferrand); Le Guern Véronique (Unité d’Hémaphérèse, Service de Médecine Interne, Hôpital Cochin, Paris); Loirat Chantal (Service de Néphrologie Pédiatrique, Hôpital Robert Debré, Paris); Moulin Bruno (Service de Néphrologie, Hôpital Civil, Strasbourg); Mousson Christiane (Service de Néphrologie, CHU de Dijon); Ojeda Uribe Mario (Service d’Hématologie, Hôpital Emile Muller, Mulhouse); Ouchenir Abdelkader (Service de Réanimation, Hôpital Louis Pasteur, Le Coudray); Parquet Nathalie (Unité de Clinique Transfusionnelle, Hôpital Cochin, Paris); Peltier Julie (Urgences Néphrologiques et Transplantation Rénale, Hôpital Tenon, Paris); Pène Frédéric (Service de Réanimation Médicale, Hôpital Cochin, Paris); Perez Pierre (Service de Réanimation polyvalente, CHU de Nancy); Poullin Pascale (Service d’hémaphérèse et d’autotransfusion, Hôpital la Conception, Marseille); Pouteil-Noble Claire (Service de Néphrologie, CHU Lyon-Sud, Lyon); Presne Claire (Service de Néphrologie, Hôpital Nord, Amiens); Provôt François (Service de Néphrologie, Hôpital Albert Calmette, Lille); Rondeau Eric (Urgences Néphrologiques et Transplantation Rénale, Hôpital Tenon, Paris); Saheb Samir (Unité d’Hémaphérèse, Hôpital la Pitié-Salpétrière, Paris); Schlemmer Benoît (Service de Réanimation Médicale, Hôpital Saint-Louis, Paris); Seguin Amélie (Service de Réanimation Médicale, centre hospitalier de Vendée); Servais Aude (Service de Néphrologie, CHU Necker-Enfants Malades); Stépanian Alain (Laboratoire d’Hématologie, Hôpital Lariboisière, Paris); Vernant Jean-Paul (Service d’Hématologie, Hôpital la Pitié-Salpétrière, Paris); Veyradier Agnès (Service d’Hématologie Biologique, Hôpital Lariboisière, Paris); Vigneau Cécile (Service de Néphrologie, Hôpital Pontchaillou, Rennes); Wynckel Alain (Service de Néphrologie, Hôpital Maison Blanche, Reims); Zunic Patricia (Service d’Hématologie, Groupe Hospitalier Sud-Réunion, la Réunion).

## Sources of Funding

This project has received funding from the Horizon 2020 Framework Programme for Research and Innovation of the European Union under Grant Agreement No. 675746.

## Disclosures

LCVP, BE, KK, MJ, SM, PP, FP, CP, TK, AS, ND, EA do not have any conflict of interest to declare. LG and YB are consultants for Sanofi. KV is a member of the advisory board of Shire-Takeda and Ablynx-Sanofi. AV is a member of the Clinical Advisory Board for Ablynx-Sanofi and Shire-Takeda. PC is a member of the Clinical Advisory Board for Alexion, Ablynx-Sanofi, Shire-Takeda and Octapharma.
