# Supplementary material for: Understanding the Health Literacy in Patients With Thrombotic Thrombocytopenic Purpura
Source: Hemasphere. 2020 Aug 11;4(4):e462. doi: 10.1097/HS9.0000000000000462 (PMC7430230; doi:10.1097/HS9.0000000000000462)
Supplement: Supplemental Digital Content [file hs9-4-e462-s001.docx]

**Understanding the health literacy in patients with Thrombotic Thrombocytopenic Purpura**

**Supplemental Tables**

**Supplemental Table 1. Scoring of answers and distribution of respondents answers to TTP literacy questions (n = 120).** The 21 questions related to aspects part of TTP literacy are depicted (Q12-Q32). Two questions were open-ended (Q15 and Q22), and from the 19 closed-ended questions; 7 were two-option response (Q12-13, Q19-Q20, Q26-27, Q29) and 12 were multiple-choice answer (Q14, Q16-Q18, Q21, Q23-Q25, Q28, Q30-Q32). Correct answers are in bold. Points attributed to each question are given (points). Number (n) and percentage (%) of patients who gave a specific answer to each question are also shown. For clarity, questions related to ‘what type of disease is TTP’ are marked in yellow, questions related to ‘which abnormalities in the body cause TTP’ in salmon, questions related to ‘what is the treatment for the disease’ in green, questions related to ‘what is the risk of relapse and what triggers TTP’ in blue.

*Without specifying which type of treatment

| Question | Points | Number of patients  n (%) |
| --- | --- | --- |
| What type of disease is TTP? |  |  |
| Q12. TTP is a contagious disease |  |  |
| No | 1 | 110 (91) |
| Yes | 0 | 1 (1) |
| Do not know | 0 | 7 (6) |
| No response | 0 | 2 (2) |
| Q13. TTP results from a reaction of the immune system against your own body | | |
| Yes | 1 | 95 (79) |
| No | 0 | 5 (4) |
| Do not know | 0 | 20 (17) |
| Q14. Thrombotic Thrombocytopenic Purpura (TTP) is |  |  |
| An autoimmune disease or a genetic disease | 1 | 6 (5) |
| An autoimmune disease | 1 | 102 (85) |
| A genetic disease | 1 | 1 (1) |
| Do not know | 0 | 8 (7) |
| No response | 0 | 3 (2) |
| Which abnormalities in the body cause TTP? | | |
| Q15. What is the missing protein in TTP? |  |  |
| ADAMTS13 | 1 | 76 (63) |
| Platelets | 0 | 3 (2) |
| Do not know | 0 | 25 (21) |
| No response | 0 | 16 (13) |
| Q16. ADAMTS13 activity in TTP is |  |  |
| Very low | 1 | 87 (73) |
| High | 0 | 5 (4) |
| Normal | 0 | 1 (1) |
| Do not know | 0 | 22 (18) |
| No response | 0 | 5 (4) |
| Q17. In TTP platelet count |  |  |
| Decreases | 1 | 109 (91) |
| Increases | 0 | 6 (5) |
| Do not know | 0 | 3 (2) |
| No response | 0 | 2 (2) |
| Q18. During TTP, the number of platelets and red blood cells are |  |  |
| Low | 1 | 104 (87) |
| Normal | 0 | 2 (2) |
| Do not know | 0 | 11 (9) |
| No response | 0 | 3 (2) |
| Q19. A deficiency of ADAMTS13 causes the formation of small blood clots | | |
| Yes | 1 | 67 (56) |
| No | 0 | 19 (16) |
| Do not know | 0 | 28 (23) |
| No response | 0 | 6 (5) |
| Q20. TTP can affect all your organs |  |  |
| Yes | 1 | 84 (70) |
| No | 0 | 24 (20) |
| Do not know | 0 | 11 (9) |
| No response | 0 | 1 (1) |
| Q21. Normally, Von Willebrand Factor |  |  |
| Prevents bleeding + is a protein of coagulation | 1 | 4 (3) |
| Is a protein of coagulation | 0.5 | 15 (13) |
| Prevents bleeding | 0.5 | 9 (7) |
| Increases bleeding | 0 | 7 (6) |
| Do not know | 0 | 79 (66) |
| No response | 0 | 6 (5) |
| What is the treatment for the disease? |  |  |
| Q22. What is the main treatment for TTP? |  |  |
| Plasma exchange | 1 | 62 (52) |
| Plasma exchange + Rituximab | 1 | 17 (14) |
| Plasma exchange + Treatment^a^ | 1 | 3 (2) |
| Plasma exchange + Corticosteroids + Rituximab | 1 | 2 (2) |
| Rituximab | 0.5 | 18 (15) |
| Corticosteroids | 0.5 | 3 (2) |
| ADAMTS13 | 0 | 1 (1) |
| Platelets | 0 | 2 (2) |
| Do not know | 0 | 8 (7) |
| No response | 0 | 4 (3) |
| Q23. Plasma exchange is efficient because it provides |  |  |
| ADAMTS13 | 1 | 59 (49) |
| Antibodies | 0 | 24 (20) |
| ADAMTS13 + Antibodies | 0 | 7 (6) |
| None of the above | 0 | 10 (8) |
| Do not know | 0 | 14 (12) |
| No response | 0 | 6 (5) |
| Q24. Plasma exchanges during the acute phase of TTP is |  |  |
| Mandatory to recover + daily | 1 | 47 (39) |
| Mandatory to recover | 0.5 | 53 (44) |
| Daily | 0.5 | 10 (9) |
| Weekly | 0 | 1 (1) |
| Do not know | 0 | 4 (3) |
| No response | 0 | 5 (4) |
| Q25. A prompt treatment of TTP |  |  |
| Improves the prognosis + should be started at day 1 | 1 | 56 (47) |
| Improves the prognosis | 0.5 | 32 (27) |
| Should be started at day 1 | 0.5 | 19 (16) |
| Can be started a couple of days later than the diagnosis | 0 | 6 (5) |
| Does not change prognosis | 0 | 1 (1) |
| Do not know | 0 | 4 (3) |
| No response | 0 | 2 (1) |
| What is the risk of relapse and what triggers TTP? |  |  |
| Q26. Do relapses occur frequently? |  |  |
| Yes | 1 | 48 (40) |
| No | 0 | 57 (47) |
| Do not know | 0 | 7 (6) |
| No response | 0 | 8 (7) |
| Q27. Rituximab reduces the risk of relapse |  |  |
| Yes | 1 | 88 (73) |
| No | 0 | 8 (7) |
| Do not know | 0 | 21 (18) |
| No response | 0 | 3 (2) |
| Q28. Rituximab is used to |  |  |
| Reduce antibodies against ADAMTS13 | 1 | 58 (48) |
| Supply ADAMTS13 | 0 | 14 (12) |
| Kill the bacteria | 0 | 6 (5) |
| Do not know | 0 | 36 (30) |
| No response | 0 | 6 (5) |
| Q29. Regular medical controls allow to minimize the risk of relapse | | |
| Yes | 1 | 108 (90) |
| No | 0 | 11 (9) |
| Do not know | 0 | 1 (1) |
| Q30. After having a TTP bout, regular medical controls are necessary | | |
| Even if you feel well | 1 | 109 (91) |
| Are not necessary | 0 | 4 (3) |
| Only if you feel sick | 0 | 2 (2) |
| Do not know | 0 | 3 (2) |
| No response | 0 | 2 (2) |
| Q31. A risk of relapse exists when the activity of ADAMTS13 is |  |  |
| Very low | 1 | 93 (78) |
| High | 0 | 6 (5) |
| Do not know | 0 | 17 (14) |
| No response | 0 | 4 (3) |
| Q32. Thrombotic Thrombocytopenic Purpura |  |  |
| Can be triggered by pregnancy + is more common in females than in males + is a rare disease | 1 | 24 (20) |
| Can be triggered by pregnancy + is more common in females than in males | 0.7 | 2 (2) |
| Is more common in females than in males + is considered a rare disease | 0.7 | 19 (16) |
| Can be triggered by pregnancy + is considered a rare disease | 0.7 | 17 (14) |
| Is more common in females than in males | 0.3 | 5 (4) |
| Is considered a rare disease | 0.3 | 43 (36) |
| Is more common in males than in females | 0 | 1 (1) |
| Do not know | 0 | 4 (3) |
| No response | 0 | 5 (4) |

^a^ Not specified

**Supplemental Table 2: Questionnaire**

The questionnaire consisted of 35 questions. In the first question (Q1), the participant’s name was requested. Forms were anonymized for analysis. The next questions were divided into three groups. The first group consisted of 10 more general questions (Q2-Q11). The second group of questions consisted of 21 questions that were designed to get insight into the patient knowledge about the disease (TTP literacy; Q12-Q32). The third group of questions contained three open-ended questions related to TTP (Q33-Q35). TTP literacy was analyzed by scoring only the second group of questions (Q12-Q32), using open-ended questions (Q15 and Q22), and closed-ended questions with two-options (Q12-13, Q19-Q20, Q26-27, Q29) and multiple-choice answers (Q14, Q16-Q18, Q21, Q23-Q25, Q28, Q30-Q32).

**Date : _________________________________________**

| 1. **Name:** | | | | | |
| --- | --- | --- | --- | --- | --- |
| 1. **Age:** | | | 1. **Gender:** | | |
| 1. **Geographic origin:** | | | 1. **Profession:** | | |
| 1. **Languages spoken fluently:** | | | | | |
| 1. **Education level** | None | Primary school | | High school | Higher education |
| 1. **When did you start to get sick?**   **_____________________________** | | | 1. **When were you diagnosed with TTP? ___________________________________** | | |
| 1. **Do you belong to a patient association?**   **_____________________________** | | | 1. **Have you used the internet site ORPHANet?**   **___________________________________** | | |

|  | | | | |  |
| --- | --- | --- | --- | --- | --- |
| 1. **TTP is a contagious disease (yes/no)** | |  | | | |
| 1. **TTP results from a reaction of the immune system against your own body (yes/no)** | |  | | | |
| 1. **Thrombotic Thrombocytopenic Purpura (TTP) is:** | | | | | |
| **an autoimmune disease** | **a genetic disease** | | **an allergy** | **a contagious disease** | |
| 1. **What is the missing protein in TTP?** | |  | | | |
| 1. **ADAMTS13 activity in TTP is** | | | | | |
| **very low** | **high** | | **normal** | **do not know** | |
| 1. **In TTP, platelet count** | | | | | |
| **increases** | **decreases** | | **is normal** | **do not know** | |
| 1. **During TTP, the number of platelets and red blood cells are** | | | | | |
| **low** | **high** | | **normal** | **do not know** | |
| 1. **A deficiency of ADAMTS13 causes the formation of small blood clots (yes/no)** | |  | | | |
| 1. **TTP can affect all your organs (yes/no)** | |  | | | |
| 1. **Normally, Von Willebrand Factor** | | | | | |
| **prevents bleeding** | **increases bleeding** | | **do not know** | **is a protein of coagulation** | |
| 1. **What is the main treatment for TTP?** | |  | | | |
| 1. **Plasma exchange is efficient because it provides (only one answer)** | | | | | |
| **ADAMTS13** | **antibodies** | | **drugs** | **none of those choices** | |
| 1. **Plasma exchanges during the acute phase of TTP are** | | | | | |
| **mandatory to recover** | **optional** | | **daily** | **weekly** | |
| 1. **A prompt treatment in TTP** | | | | | |
| **improves the prognosis** | **does not change prognosis** | | **should be started at day 1** | **can be started a couple of days later than the diagnosis** | |
| 1. **Do relapses occur frequently?* (yes/no)** | | |  | | |
| 1. **Rituximab reduces the risk of relapse (yes/no)** | | |  | | |
| 1. **Rituximab is used to** | | | | | |
| **reduce antibodies against ADAMTS13** | **supply ADAMTS13** | | **kill the bacteria** | **do not know** | |
| 1. **Regular medical controls allow to minimize the risk of relapse (yes/no)** | | |  | | |
| 1. **After having a TTP bout, regular medical controls are necessary** | | | | | |
| **only if you feel sick** | **even if you feel healthy** | | **are not necessary** | **do not know** | |
| 1. **A risk of relapse exists when the activity of ADAMTS13 is** | | | | | |
| **very low** | **high** | | **normal** | **do not know** | |
| 1. **Thrombotic Thrombocytopenic Purpura (TTP)** | | | | | |
| **is more common in males than in females** | **can be triggered by pregnancy** | | **is more common in females than in males** | **is considered as a rare disease** | |
| 1. **If you are under the impression of having a relapse with red spots on your skin or you are unusually tired, what do you do?**   **What do you do if you feel numbness in your arm or in a part of your face?** | | | | | |
| 1. **Is there something that seems unclear for you and what would you like to have clarified related to your disease?** | | | | | |
| 1. **Do you have any questions?** | | | | | |

*The term frequent was exposed to the patients as enough to need regular follow-up in order to prevent relapses
